# An integrative analysis of DNA methylation and gene expression to predict lung adenocarcinoma prognosis

**DOI:** 10.3389/fgene.2022.970507

**Published:** 2022-08-29

**Authors:** Liexi Xu, Zhengrong Huang, Zihang Zeng, Jiali Li, Hongxin Xie, Conghua Xie

**Affiliations:** ^1^ Department of Radiation and Medical Oncology, Wuhan University of Zhongnan Hospital, Wuhan, China; ^2^ Tumor Precision Diagnosis and Treatment Technology and Translational Medicine, Hubei Engineering Research Center, Zhongnan Hospital of Wuhan University, Wuhan, China; ^3^ Hubei Key Laboratory of Tumor Biological Behaviors, Zhongnan Hospital of Wuhan University, Wuhan, China

**Keywords:** DNA methylation, integrative analysis, lung adenocarcinoma, prognosis, TCGA

## Abstract

**Background:** Abnormal DNA methylation of gene promoters is an important feature in lung adenocarcinoma (LUAD). However, the prognostic value of DNA methylation remains to be further explored. *Objectives.* We sought to explore DNA methylation characteristics and develop a quantifiable criterion related to DNA methylation to improve survival prediction for LUAD patients.

**Methods:** Illumina Human Methylation450K array data, level 3 RNA-seq data and corresponding clinical information were obtained from TCGA. Cox regression analysis and the Akaike information criterion were used to construct the best-prognosis methylation signature. Receiver operating characteristic curve analysis was used to validate the prognostic ability of the DNA methylation-related feature score. qPCR was used to measure the transcription levels of the identified genes upon methylation.

**Results:** We identified a set of DNA methylation features composed of 11 genes (*MYEOV*, *KCNU1*, *SLC27A6*, *NEUROD4*, *HMGB4*, *TACR3*, *GABRA5*, *TRPM8*, *NLRP13*, *EDN3* and *SLC34A1*). The feature score, calculated based on DNA methylation features, was independent of tumor recurrence and TNM stage in predicting overall survival. Of note, the combination of this feature score and TNM stage provided a better overall survival prediction than either of them individually. The transcription levels of all the hypermethylated genes were significantly increased after demethylation, and the expression levels of 3 hypomethylated proteins were significantly higher in tumor tissues than in normal tissues, as indicated by immunohistochemistry data from the Human Protein Atlas. Our results suggested that these identified genes with prognostic features were regulated by DNA methylation of their promoters.

**Conclusion:** Our studies demonstrated the potential application of DNA methylation markers in the prognosis of LUAD.

## 1 Introduction

Lung adenocarcinoma (LUAD) is the most common histological subtype of lung cancer, accounting for approximately 50% of all lung cancer cases in most countries (Goldstraw et al., 2011; Bray et al., 2018). Previous studies have revealed that in addition to cigarette smoking, risk factors such as age, environmental pollution, occupational exposure, race, sex, and preexisting lung disease are also substantially involved in lung cancer. With the development and popularization of public databases in recent years, an increasing number of researchers have tried to identify prognostic biomarkers for LUAD by analyzing clinical characteristics and molecular information (Edge and Compton, 2010). The TNM staging system of the American Joint Commission on Cancer (AJCC) was reported to have great value in LUAD prognosis (Folkman, 1971). Liu et al. stated that conventional staging alone was not enough to predict prognosis and guide treatment decisions. They analyzed large cohorts from The Cancer Genome Atlas (TCGA) database and developed a 4-gene feature related to glycolysis (Kaishang et al., 2018). Su et al. identified an RNA sequencing network of 29 key lncRNAs, 72 mRNAs and 24 miRNAs as potential biomarkers to optimize the diagnosis and prognosis of LUAD patients by using the TCGA database (Li et al., 2017). The findings from these studies indicate that it is feasible to use different molecular markers and clinical features in public databases to establish practical models that have great application potential. Although the effectiveness of these prediction models has not been tested in clinical practice, it is necessary to continue to mine and improve the gene signatures related to the prognosis of LUAD.

Epigenetic disorders, especially abnormal DNA methylation in gene promoters, are a fundamental feature of human malignant tumors (Liu et al., 2019). As one of the most well-studied epigenetic modifications, DNA methylation mainly occurs at 5′-cytosine-phosphate-guanine-3' (CpG) dinucleotides and is regulated by DNA methyltransferases and DNA demethylases (Yang et al., 2022). Methylation and cancer formation are associated in 2 main ways: one is the regulation of tumor suppressor gene expression by gene hypermethylation in the promoter, and the other is genome-wide hypomethylation, which plays an important role in the stability of the heterochromatin structure (Feinberg, 2007). In virtually every step of tumor progression, there is abnormal promoter methylation regulation (Sui et al., 2016). *APC*, *CDH13*, *MLH1* and *IRX1* have hypermethylation in promoter CpG islands (CGIs). The hypermethylation of the *APC* and *CDH13* genes in LUAD is associated with cancer cell adhesion, and the loss of *MLH1* and *IRX1* expression is associated with poor tumor survival (Goto et al., 2009; Küster et al., 2020). The hypomethylation of *LINE-1* and *ELF3* induces protein overexpression in LUAD. The overexpression of *ELF3* can stimulate the carcinogenic phenotype of LUAD cells and reduce the survival time of patients, suggesting that the hypomethylation of *LINE-1* is a prognostic marker of LUAD development and progression (Ikeda et al., 2013; Enfield et al., 2019).

TCGA has disclosed the clinical information of more than 10,000 patients and the molecular phenotype information of their tumor tissues. This information covers 33 different types of tumors and multiple data from different sources, including transcriptomic, methylomic and proteomic sources (Mazor et al., 2016). By integrating data from different sources, we can identify specific events in the carcinogenic process and identify potential biomarkers associated with patient survival.

In this study, we obtained the TCGA Illumina Human Methylation 450K microarray data, RNA-seq data, and clinical data of LUAD patients and performed an integrative analysis to identify a set of DNA methylation features for 11 genes. We performed area under the receiver operating characteristic curve (AUC-ROC) analysis to verify the ability of the identified DNA methylation feature to predict the survival of LUAD. In addition, we performed qPCR, and the results suggested that these identified genes with prognostic features were regulated by DNA methylation in their promoters.

## 2 Materials and methods

### 2.1 Data preparation

**FIGURE 1:**
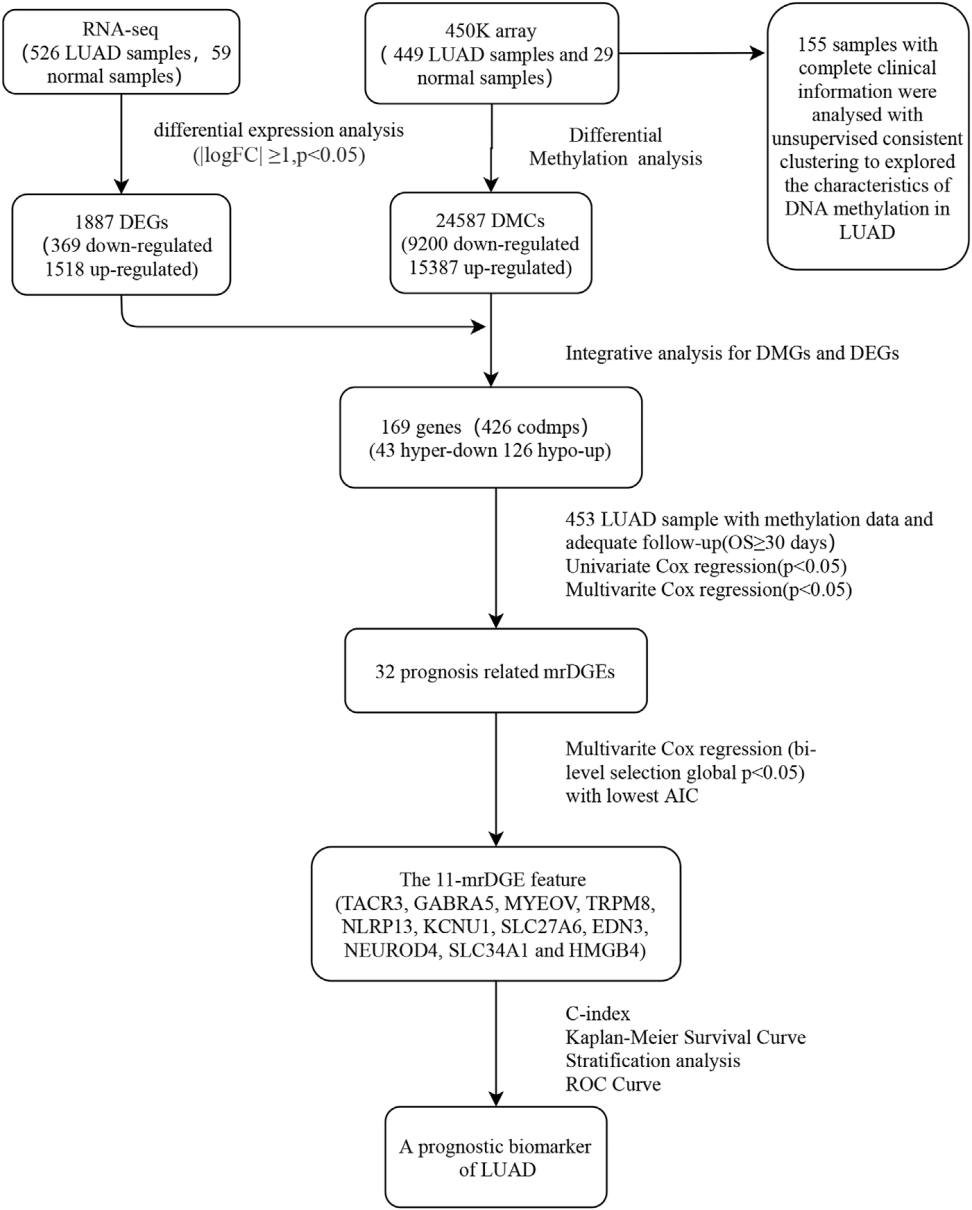
Flow chart for obtaining and verifying methylation feature.

The steps of data acquisition and analysis, as well as methylation feature acquisition and verification, are shown in the flow chart (Figure 1). Illumina Human Methylation 450K array data were obtained from TCGA, and a total of 24,587 DMSs from 478 pretreated methylation arrays were screened using the camp and Minfi R software packages. After that, 478 samples (449 LUAD samples and 29 normal samples) were included after being filtered, inspected, and standardized with the ChAMP R package. Level 3 RNA-seq data from TCGA were normalized and log2 transformed by the edgeR package. For the preprocessing of clinical information corresponding to the sample, patients with nonsurvival status or survival time less than 1 month were excluded because of other disease-related deaths.

### 2.2 Differential methylation analysis and differential expression analysis

In total, 449 LUAD samples and 29 normal samples were subjected to differential methylation analysis with the ChAMP R package and the Minfi R package (Li et al., 2019). Principal component analysis was used to detect the sample quality. The ChAMP DMP function and the Minfi R package defined the methylation loci with an average methylation difference >0.2 and a false discovery rate <0.05 as differentially methylated sites (DMSs), and the final DMSs were obtained through the intersection of the two (Li et al., 2018). Differentially expressed genes (DEGs) between the 526 LUAD samples and the 59 normal samples were analyzed with the Limma R package (*p* < 0.05 and |log2FC| ≥ 1). Metascape (https://metascape.org/gp/index.html#/main/step1) was used to analyze the pathway enrichment of the hyper-down and hypo-up methylated related differential expression genes (mrDEGs) groups.

### 2.3 Survival model construction process

A prognosis prediction model was established according to the DNA methylation β value of mrDEGs and matched prognostic data of patients. According to the methylation β value, univariate Cox regression analysis was used to screen mrDEGs (*p* < 0.01) that were significantly associated with overall survival (OS). Then, mrDEGs identified in the univariate Cox regression analysis were subjected to multivariate Cox regression analysis (Lian et al., 2019). At the same time, the Akaike information criterion (AIC) was used to screen out the genes with subtly individual but significantly synergistic effects to determine the most appropriate gene feature (Tozzi et al., 2020). A Kaplan‒Meier (K-M) curve with a log rank test was used to validate the survival difference of patients (Zhao et al., 2020). Harrell’s concordance index (C-index) and the corresponding 95% confidence intervals (CIs) were calculated to determine the prognostic model’s ability. These steps were performed by R with the survival and survcomp R packages (Schröder et al., 2011).

### 2.4 Consensus clustering analysis

We selected 155 LUAD samples with complete clinical information. For our standard, we considered the standard deviation of the β value in tumor samples to be greater than 0.2 and the average β value in normal tissues to be less than 0.05. With this approach, we selected 641 methylation probes according to the standard. According to the PAM algorithm and Euclidean distance, we then performed unsupervised consistent clustering on 641 probes of 155 samples. The Consensus Cluster Plus R package was used for the clustering analysis (Wilkerson and Hayes, 2010). The Kruskal‒Wallis test was used to validate the significance of clinical features among clusters.

### 2.5 Validation experiments in cell lines

qPCR was used to verify the changes in gene transcription levels upon methylation. A549, PC9 and H1975 cells were purchased from the Canadian Standards Association (CSA). All cell lines were cultured in RPMI 1640 with 10% fetal bovine serum. All experimental cells were treated with 5-Aza-2′- deoxycytidine (5-aza, Aladdin) for 96 h at 1 µM. qPCR analyses of all cell lines were repeated at least 3 times (Christman, 2002). All primer sequences used in qPCR are listed in Attachment 1: Table 1.

**TABLE 1:** All primer sequences used in qPCR.

Primer name	5′ Sequence 3′
EDN3 Fp	ATT​GCC​ACC​TGG​ACA​TCA​TT
EDN3 Rp	GCA​GGC​CTT​GTC​ATA​TCT​CC
TACR3 Fp	TTC​ATC​CAA​ACC​GGC​AAA​GC
TACR3 Rp	AAA​CTT​GGG​TCT​CTT​GGC​GT
SLC27A6 Fp	AAAAAGGGGGACACGGTG
SLC27A6 Rp	AGGAGGGAGTTGGAGCGA

### 2.6 Statistical analysis

The correlation between feature scores and clinical factors was analyzed by the chi square or Fisher exact test (Jung, 2014; Pandis, 2016). Multivariate Cox regression combined with hierarchical data analysis was used to evaluate the predictive power of the clinical features, TNM stage, and methylation feature score for prognosis. The forest map was drawn by Prism, and other statistical tests were performed by R using the corresponding R packages.

## 3 Results

### 3.1 Differential methylation and the identification of mrDMGs

**FIGURE 2:**
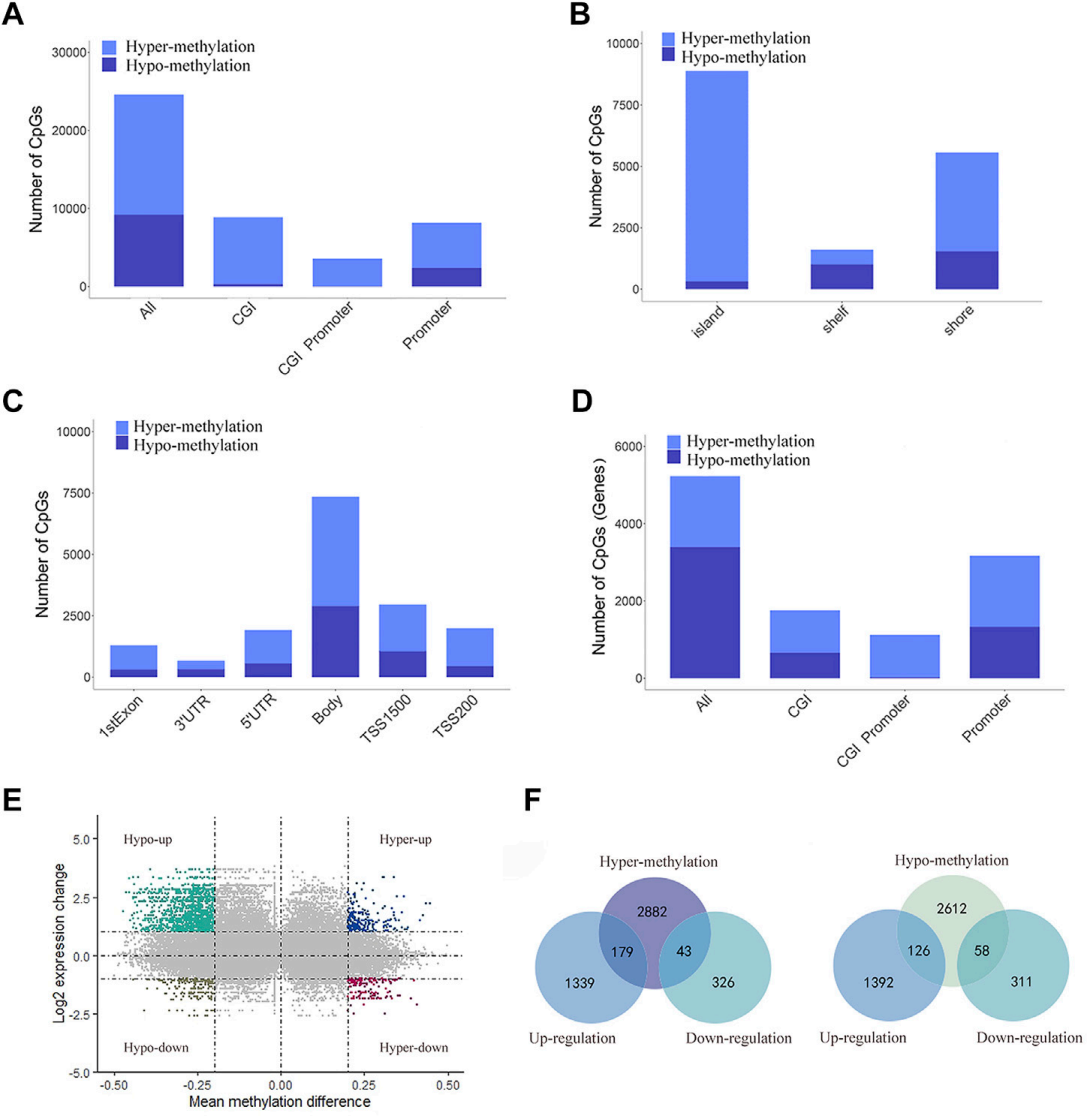
Distribution of DMSs and obtain mrDEGs of LUAD. **(A)** Distribution of DMSs across various genomic regions, including CpG islands (CGI), promoters, CGI promoter, and the whole genome (all). **(B)** Distribution of DMSs in various areas related to CGI distance, including CpG shelves, CpG shores and CpG islands. **(C)** Distribution of DMSs in gene location, including 3′ UTRs, gene bodies, first exons, 5′ UTRs, TSS200 and TSS1500. **(D)** Distribution of DMGs across various genomic regions. **(E)** Scatter plot shows mean methylation difference versus log2 expression change, and each point represents a pair of methylation site and gene. **(F)** Venn diagrams shows the intersection between DEGs and hypermethylated genes (left) and between DEGs and hypomethylated genes (right).

Illumina Human Methylation 450K array data were obtained from TCGA. We screened a total of 24,587 DMSs from data from 478 pretreated methylation arrays using the camp and Minfi R software packages. We then divided DMSs into 15,387 hypermethylated and 9,200 hypomethylated sites and evaluated their distribution in the genome. Compared to 63% in the whole genome, hypermethylated sites increased significantly in the promoter, CGIs, and CGI promoters (71%, 96% and 99%, respectively, Figure 2A). At the same time, most DMSs on CGIs were hypermethylated (96%), and most DMSs on shelf CpG positions were hypomethylated (Figure 2B). When we detected the distribution of DMSs around the gene, we found that the hypermethylation of CpGs was higher near the transcription start site (TSS). For example, the proportions of hypermethylated CpGs in the 5′UTR, tss200 and first exon were 70%, 77%, and 76%, respectively (Figure 2C). Next, we located DMSs on the gene and obtained 5,900 differentially methylated genes (DMGs) (Figure 2D). Next, we identified 1,887 DEGs based on the RNA-seq data. Then, 406 mrDEGs were determined through the intersection of the DMGs and the identified DEGs (Figure 2E). Among them, 43 mrDEGs were in the hypermethylation downregulation group (hyper-down group), and 126 mrDEGs were in the hypomethylation upregulation group (hypo-up group) (Figure 2F).

### 3.2 mrDEGs involved in biological processes

**FIGURE 3:**
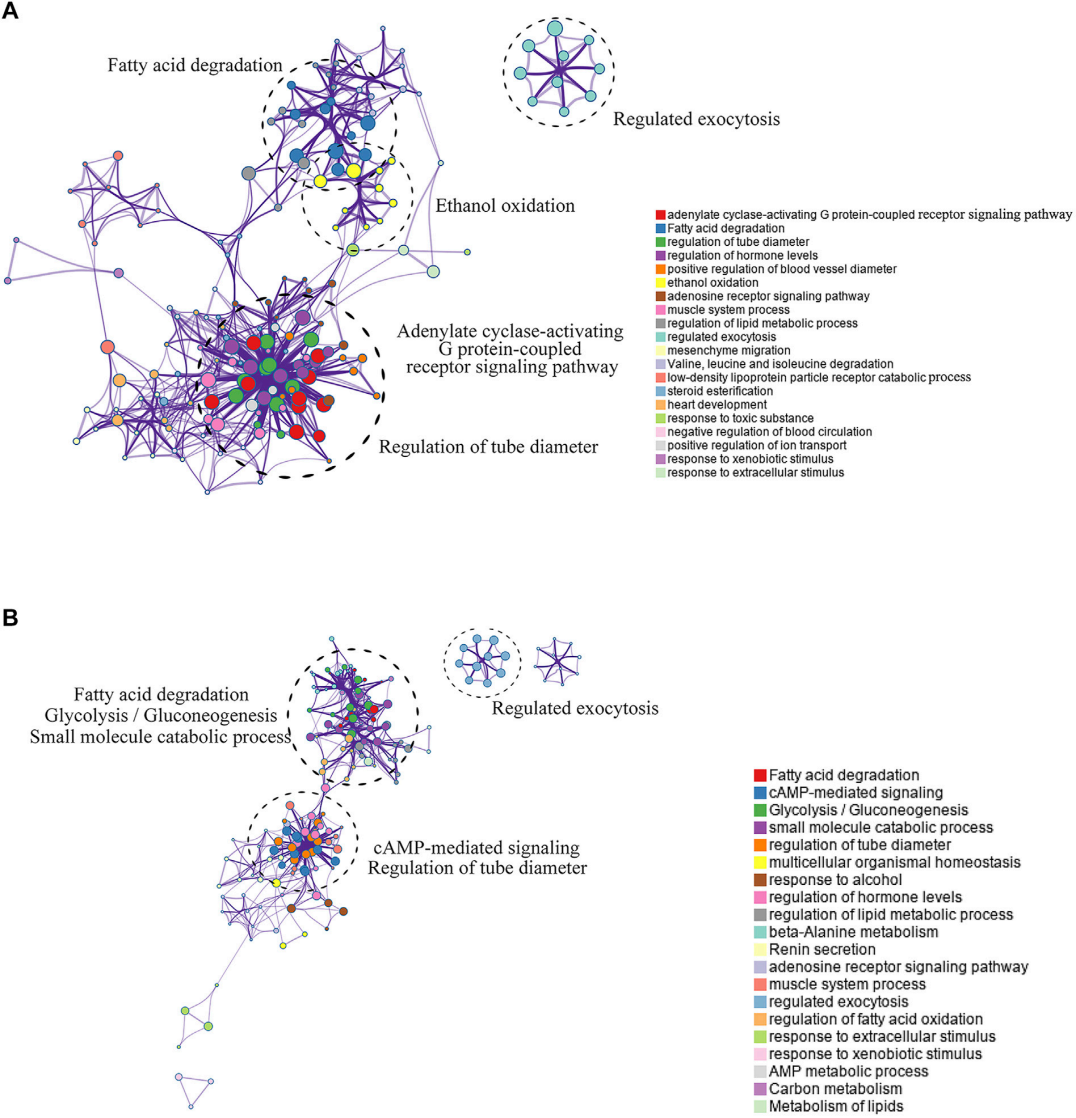
Pathway enrichment analysis of mrDEGs in LUAD. **(A)** The pathway enrichment analysis of the upregulated mrDEGs. Each node represents a gene group. The node size is proportional to the total number of genes in each gene set. The width of the line between nodes represents the proportion of genes shared among gene sets. **(B)** The pathway enrichment analysis of the downregulated mrDEGs.

The Metascape website was used to analyze the pathway enrichment of the hyper-down and hypo-up mrDEGs. In the hypo-up group, the genes showed a significant abundance in fatty acid degradation, cyclic adenosine monophosphate (cAMP)-mediated signaling, glycolysis/gluconeogenesis, etc. (Figure 3A). Cancer is usually accompanied by nutritional metabolic imbalances, such as abnormal glucose and lipid metabolism (Li and Liao, 2021). cAMP was the first second messenger to be discovered, and it plays key roles in physiological defects caused by metabolic disorders (Zhang et al., 2020; Chi et al., 2021). Interestingly, in the hyper-down group, there was also gene enrichment related to fatty acid degradation (Figure 3B). The effects of lipid metabolism disorders on cancer have attracted increasing attention in recent years (Karagiota et al., 2022). There is no doubt that to reprogram their metabolic state and ensure cell survival, tumor cells need epigenetic modifications to regulate gene expression. For example, brother of the regulator of imprinted sites can mediate the Warburg effect and promote breast cancer by regulating the methylation of pyruvate kinase M1/2 (PKM) exons (Singh et al., 2017; Huo et al., 2021). Our results suggest that abnormal lipid metabolism in LUAD may be closely mediated by DNA methylation (Figure 3B).

### 3.3 Identification of prognostic mrDEGs

We established a prognosis prediction model according to the methylation data of mrDEGs and the matched prognostic data. We first analyzed 406 mrDEGs in 453 patients by univariate Cox regression and identified 32 mrDEGs related to prognosis (*p* < 0.05). Next, multivariate Cox regression analysis was performed on the 32 mrDEGs. The AIC, as the indicator for model fitness, determined the most suitable prognostic model. Finally, we identified 11 mrDEGs (*MYEOV*, *KCNU1*, *SLC27A6*, *NEUROD4*, *HMGB4*, *TACR3*, *GABRA5*, *TRPM8*, *NLRP13*, *EDN3* and *SLC34A1*) to be included in a DNA methylation feature prognostic model. There were 5 genes (*MYEOV*, *KCNU1*, *SLC27A6*, *NEUROD4* and *HMGB4*) with statistically nonsignificant *p* values in multivariate Cox regression analysis (Table 2). However, the AIC of this prognostic model was the lowest (AIC = 1733.8, p = 2e-05), indicating that this model was the most suitable, and the overall effect of the model was significant. The C-index of the identified DNA methylation feature model was 0.666 (95% CI = 0.641–0.690), indicating great discrimination ability. The correlation between methylation level and gene expression of these 11 genes is shown in the appendix (Supplementary Figure.S1).

**TABLE 2:** Eleven mrDEGs identified as a DNA methylation signature prognostic model.

Gene symbol	Full name	Chr	Coefficient	*p* value
TACR3	Tachykinin receptor 3	4q24	2.513	0.049
GABRA5	Gamma-aminobutyric acid type A receptor subunit Alpha5	15q12	1.74	0.028
MYEOV	Myeloma overexpressed	11q13.3	−0.752	0.147
TRPM8	Transient receptor potential cation channel subfamily M member 8	2q37.1	−1.611	0.008
NLRP13	NLR family pyrin domain containing 13	19q13.43	−2.074	0.01
KCNU1	Potassium calcium-activated channel subfamily U member 1	8p11.23	−1.158	0.072
SLC27A6	Solute carrier family 27 member 6	5q23.3	−1.694	0.398
EDN3	Endothelin 3	20q13.32	−2.059	0.01
NEUROD4	Neuronal differentiation 4	12q13.2	−1.243	0.098
SLC34A1	Solute carrier family 34 member 1	5q35.3	1.626	0.004
HMGB4	High mobility group box 4	1p35.1	0.91	0.138

### 3.4 DNA methylation feature model for predicting the OS of LUAD patients

According to the correlation coefficients of the eleven mrDEGs obtained by multivariate Cox regression analysis, we established a feature score formula.

$$\begin{array}{l} \text{feature score }=\left(2.513\text{ ∗ }methylation\;\beta \;value\;of\;TACR3\right)\\ \;\;\;\;\;\;\;\;\;\;\;\;\;\;\;\;\;\;\;\;\;\;\;\;\;\;\;\;+\;\left(1.740\text{ ∗ }methylation\;\beta \;value\;of\;GABRA5\right)\\ \;\;\;\;\;\;\;\;\;\;\;\;\;\;\;\;\;\;\;\;\;\;\;\;\;\;\;\;+\;\left(-0.752\text{ ∗ }methylation\;\beta \;value\;of\;MYEOV\right)\\ \;\;\;\;\;\;\;\;\;\;\;\;\;\;\;\;\;\;\;\;\;\;\;\;\;\;\;\;+\;\left(-1.611\text{ ∗ }methylation\;\beta \;value\;of\;TRPM8\right)\\ \;\;\;\;\;\;\;\;\;\;\;\;\;\;\;\;\;\;\;\;\;\;\;\;\;\;\;\;+\;\left(-2.074\text{ ∗ }methylation\;\beta \;value\;of\;NLRP13\right)\\ \;\;\;\;\;\;\;\;\;\;\;\;\;\;\;\;\;\;\;\;\;\;\;\;\;\;\;\;+\;\left(-1.158\text{ ∗ }methylation\;\beta \;value\;of\;KCNU1\right)\\ \;\;\;\;\;\;\;\;\;\;\;\;\;\;\;\;\;\;\;\;\;\;\;\;\;\;\;\;+\;\left(-1.694\text{ ∗ }methylation\;\beta \;value\;of\;SLC27A6\right)\\ \;\;\;\;\;\;\;\;\;\;\;\;\;\;\;\;\;\;\;\;\;\;\;\;\;\;\;\;+\;\left(-2.059\text{ ∗ }methylation\;\beta \;value\;of\;EDN3\right)\\ \;\;\;\;\;\;\;\;\;\;\;\;\;\;\;\;\;\;\;\;\;\;\;\;\;\;\;\;+\;\left(-1.243\text{ ∗ }methylation\;\beta \;value\;of\;NEUROD4\right)\\ \;\;\;\;\;\;\;\;\;\;\;\;\;\;\;\;\;\;\;\;\;\;\;\;\;\;\;\;+\;\left(1.626\text{ ∗ }methylation\;\beta \;value\;of\;SLC34A1\right)\\ \;\;\;\;\;\;\;\;\;\;\;\;\;\;\;\;\;\;\;\;\;\;\;\;\;\;\;\;+\;\left(0.910\text{ ∗ }methylation\;\beta \;value\;of\;HMGB4\right) \end{array}$$

**FIGURE 4:**
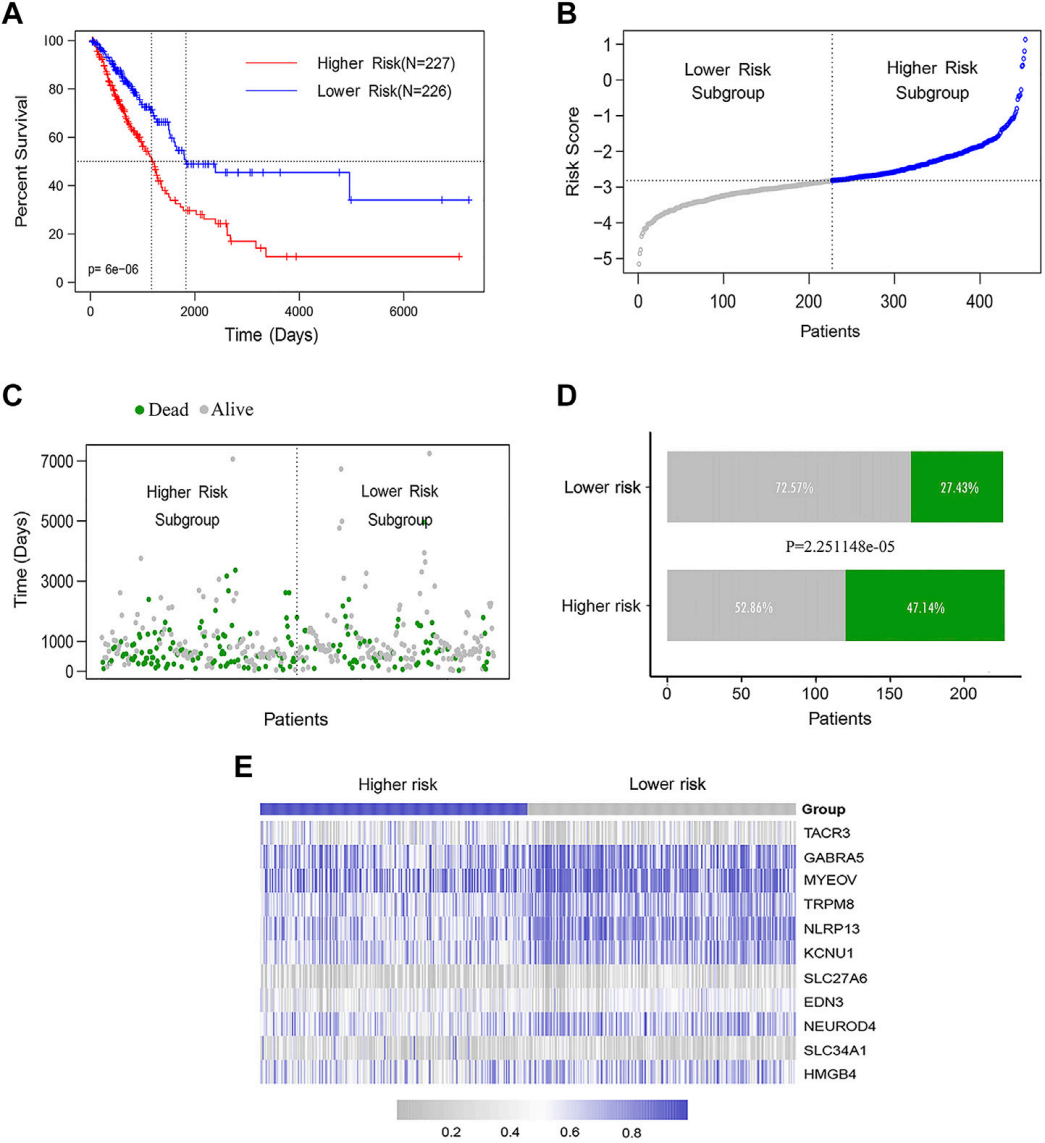
The DNA methylation feature for predicting OS prediction in LUAD patients. **(A)** K-M assessed OS based on the DNA methylation feature. The LUAD patients were divided into the lower-risk (*n* = 226) and higher-risk (n = 227) subgroups according to the median of the methylation scores. Log rank test was used between curves (*p* < 0.0001). **(B)** The distribution of feature scores for DNA methylation feature of patients. **(C–D)** The distribution of survival status of LUAD patients in the lower- and higher-risk groups (Chi-square test, *p* < 0.0001). **(E)** The methylation β value spectrum of 11 DNA methylation feature genes.

The LUAD patients were ranked according to their calculated methylation-related feature scores and divided into higher-risk (n = 226) and lower-risk groups (n = 227) according to the median. The K-M curve showed that the median OS of the higher-risk group was significantly shorter than that of the lower-risk group (log rank test *p* < 0.0001) (Figure 4A). We also analyzed the distribution of the methylation feature scores, patient survival statuses and methylated β values in LUAD patients, as well as the methylation β value spectra of 11 DNA methylation feature genes (Figures 4B–E).

### 3.5 DNA methylation feature model with clinicopathological features

**FIGURE 5:**
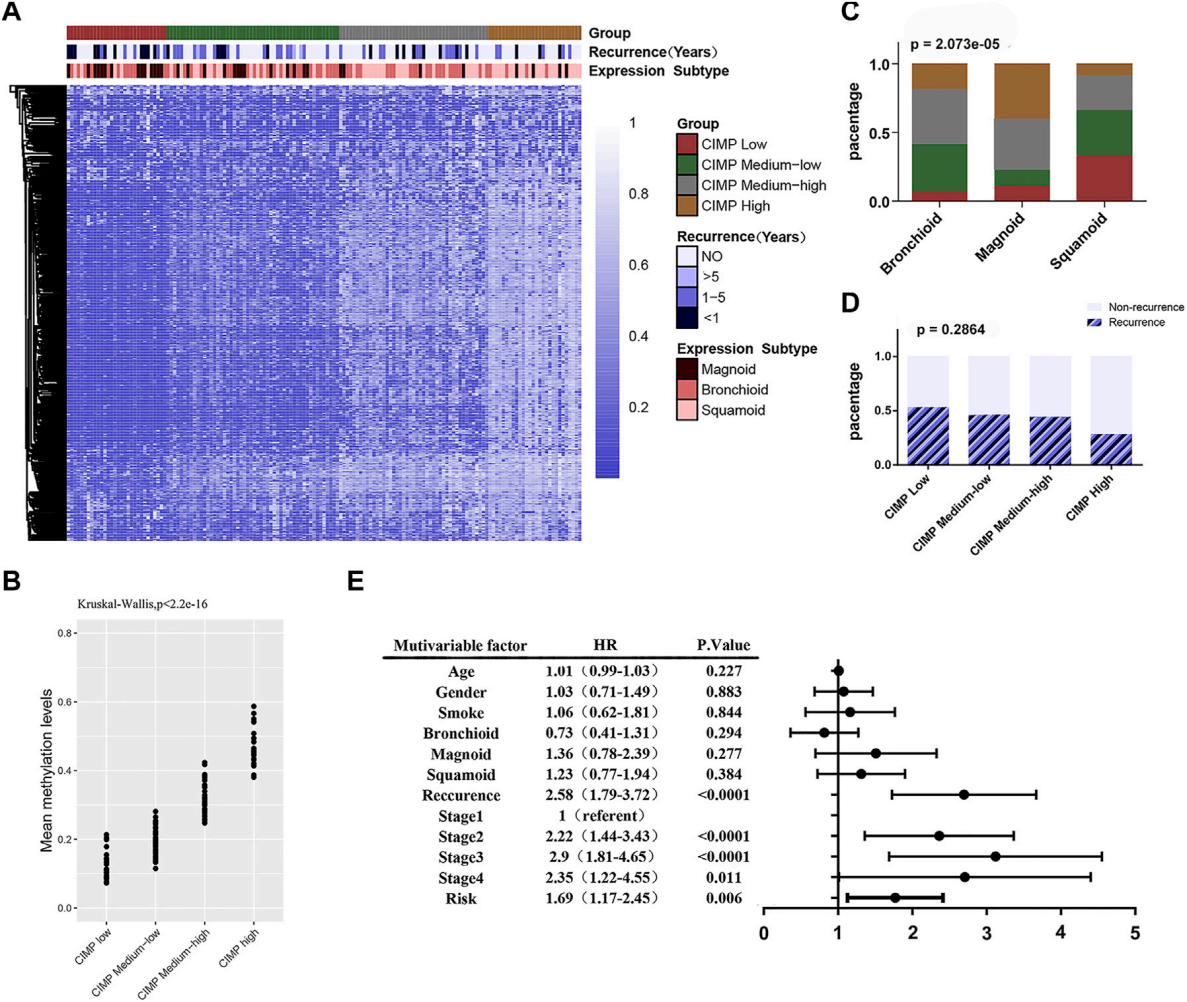
The DNA methylation feature with clinicopathological features. **(A)** Unsupervised cluster analysis of the methylation levels in LUAD. A total of 155 samples are presented in rows, and 641 CpG loci with the largest variation (mean methylation level β < 0.05 in normal samples and standard deviation σ > 0.20 in tumor samples) are listed. The 4 identified clusters are represented as CIMP high (n = 28), CIMP medium high (n = 45), CIMP medium low (n = 52) and CIMP low (*n* = 30). **(B)** Significant differences (*p* < 0.0001) in the methylation levels of the 4 clusters. **(C)** The sample distributions in terms of tumor subtype (Chi-square test, *p* < 0.0001). **(D)** The sample distributions in terms of recurrence (Chi-square test, *p* = 0.2864). **(E)** Forest map: multivariate Cox regression analysis was used to analyze the prognostic values of age, gender, smoking, tumor expression subtype, tumor recurrence, TNM stage, feature score and other clinicopathological features in 391 cases.

First, we attempted to validate the correlation between DNA methylation levels and clinicopathological features in LUAD. We then performed unsupervised consistent clustering of the 641 most variable DNA methylation probes in 155 samples (with complete clinical information) into 4 clusters: CGI Methylator Phenotype (CIMP) high, CIMP medium high, CIMP medium low and CIMP low (Figure 5A). The average methylation levels among the different clusters were significant (*p* < 2.2e-16) (Figure 5B). DNA methylation was significantly correlated with tumor subtype (X-squared = 31.457, *p* = 2.073e-05) (Figure 5C). Most of magnoid tumors were enriched in the CIMP low and CIMP medium low clusters, while the CIMP high group had more squamoid tumors Furthermore, DNA methylation showed a trend related to tumor recurrence; however, there was no statistical Figure 5A Significance (*p* = 0.2864) (Figure 5D).

Then, we analyzed the correlation between DNA methylation feature scores and clinicopathological features. The results showed that the methylation feature score was significantly correlated with tumor subtype (Table 3, *p* = 0.032). In addition, the DNA methylation feature score was associated with smoking history in LUAD patients (Table 3, *p* = 0.004). Previous studies have shown that smoking is associated with methylation levels. For example, hypomethylation at cg05575921 in the aryl hydrocarbon receptor repressor gene was strongly associated with the smoking behavior of an individual (Jamieson et al., 2020). Therefore, the hypothesis that prognostic signals are related to smoking is reasonable. To delve into the effects of DNA methylation and clinicopathological features on prognosis, 391 patients with complete clinicopathological features were analyzed in a Cox regression model. The forest map showed that the feature score (HR = 1.69, 95% CI = 1.17–2.45, *p* = 0.006), the TNM staging system, and tumor recurrence (HR = 2.58, 95% CI = 1.79–3.72, *p* < 0.0001) were independent prognostic factors for LUAD, while smoking history and tumor subtype were not (Figure 5E). DNA methylation is closely related to tumor immune microenvironment (Chiappinelli and Baylin, 2022; Li et al., 2022). We investigated the relationship between methylation feature scores and tumor immune infiltration. In the group with low methylation feature scores, we observed an increase in monocyte, dendritic cell (resting) and mast cell (resting) infiltration, as well as a decrease in macrophage (M0) cell infiltration. However, there is no changes of immune effector cells observed (Supplementary Figure S2).

**TABLE 3:** The correlation between DNA methylation feature scores and clinicopathological feature.

	N	High	Low	P
Age (years)	453			
≥ 60	313	147 (46%)	166 (54%)	0.098
< 60	130	73 (56%)	57 (44%)	
Sex	453			
female	239	109 (46%)	130 (54%)	0.067
male	214	117 (55%)	97 (45%)	
Tumor location	440			
right	257	126 (49%)	131 (51%)	0.784
left	183	93 (51%)	90 (49%)	
T stage	453			
TX+T1+T2	398	200 (50%)	198 (50%)	0.787
T3+T4	55	26 (47%)	29 (53%)	
N stage	452			
N0	299	145 (48%)	154 (52%)	0.427
N1+N2+N3	153	81 (53%)	72 (47%)	
M stage	448			
M0	428	216 (50%)	212 (50%)	1
M1	20	10 (50%)	10 (50%)	
TNM stage	448			
I+II	350	175 (50%)	175 (50%)	0.949
III+IV	98	50 (51%)	48 (49%)	
Recurrence	416			
YES	162	85 (52%)	77 (48%)	0.39
NO	254	121 (48%)	133 (52%)	
Subtype	193			
Bronchioid	69	24 (35%)	45 (65%)	0.032
Magnoid	49	28 (57%)	21 (43%)	
Squamoid	75	39 (52%)	36 (48%)	
Smoke	430			
Non-smoker	63	20 (32%)	43 (68%)	0.004
Current smoker	367	192 (52%)	175 (48%)	

High and low groups were divided according to median of feature scores.

### 3.6 Prognostic value of the DNA methylation feature score is independent of TNM stage and cancer recurrence

**FIGURE 6:**
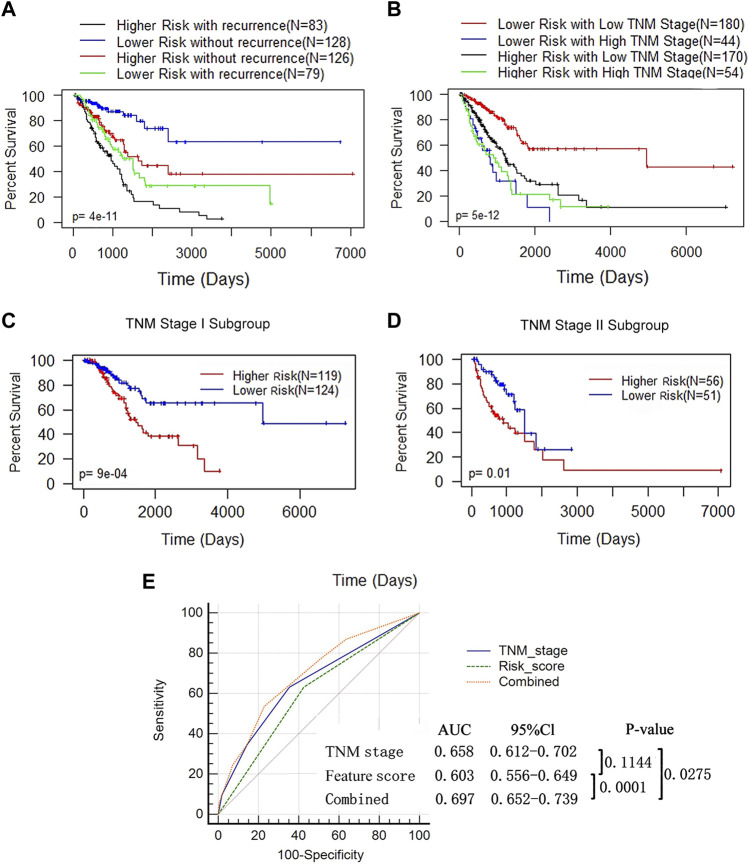
The prognostic value of DNA methylation feature score was not associated with tumor recurrence status and TNM stage. **(A)** K-M analysis of OS based on the DNA methylation feature and recurrence status. The LUAD patients were divided into the lower- and higher-risk subgroups according to the median of the methylation feature scores, with or without recurrence. Log-rank test (*p* < 0.0001). **(B)** K-M analysis of OS based on the DNA methylation feature and TNM stage. The LUAD patients were divided into the lower- and higher-risk subgroups according to the median of the methylation feature scores, and divided into the low (stage I+ II, n = 350) and high (stage III+IV, *n* = 98) stages according to the TNM stage (Log-rank test, *p* < 0.0001). **(C)** K-M curves for patients in the TNM stage I subgroup (*n* = 350). **(D)** K-M curves for patients in the TNM stage II subgroup (*n* = 98). **(E)** ROC analysis assessed the sensitivity and specificity of DNA methylation feature score, TNM stage and the combination of the 2 factors in predicting OS.

Since a high feature score, tumor recurrence and a high TNM stage were independent adverse prognostic factors for LUAD (Figure 5D), we performed a combined analysis between DNA methylation features and the other 2 influencing factors. We found that the prognosis of patients in the higher-feature score group was poorer, whether in the recurrence (log rank test, *p* < 0.0009) or nonrecurrence subgroup (log rank test, *p* = 0.02) (Figure 6A). In the combined analysis of TNM stages and DNA methylation features, we found that patients in the lower TNM stage (I and II) subgroups had a notably worse prognosis when they were also in the high-feature score subgroups (p = 2e-05) but not in the higher TNM stage (III and IV) subgroups (*p* = 0.3) (Figure 6B). We further classified patients at low TNM stages and found that the *p* value of the K-M curve in the stage I subgroup (p = 9e-04) was more significant than that in the stage II subgroup (*p* = 0.01) (Figures 6C,D). These results suggested that the DNA methylation feature score was more valuable in patients at a lower TNM stage. AUC-ROC analysis was used to evaluate the sensitivity and specificity of the prediction model (Figure 6E). The combination of this feature score and TNM stage was significantly superior to that of TNM stage alone (0.697 vs 0.658, *p* = 0.0275) or feature score alone (0.697 vs 0.603, *p* = 0.0001). These results suggested that the combination of the DNA methylation feature score and TNM stage might help to improve OS prediction in LUAD patients.

### 3.7 The expression of the eleven identified genes

**FIGURE 7:**
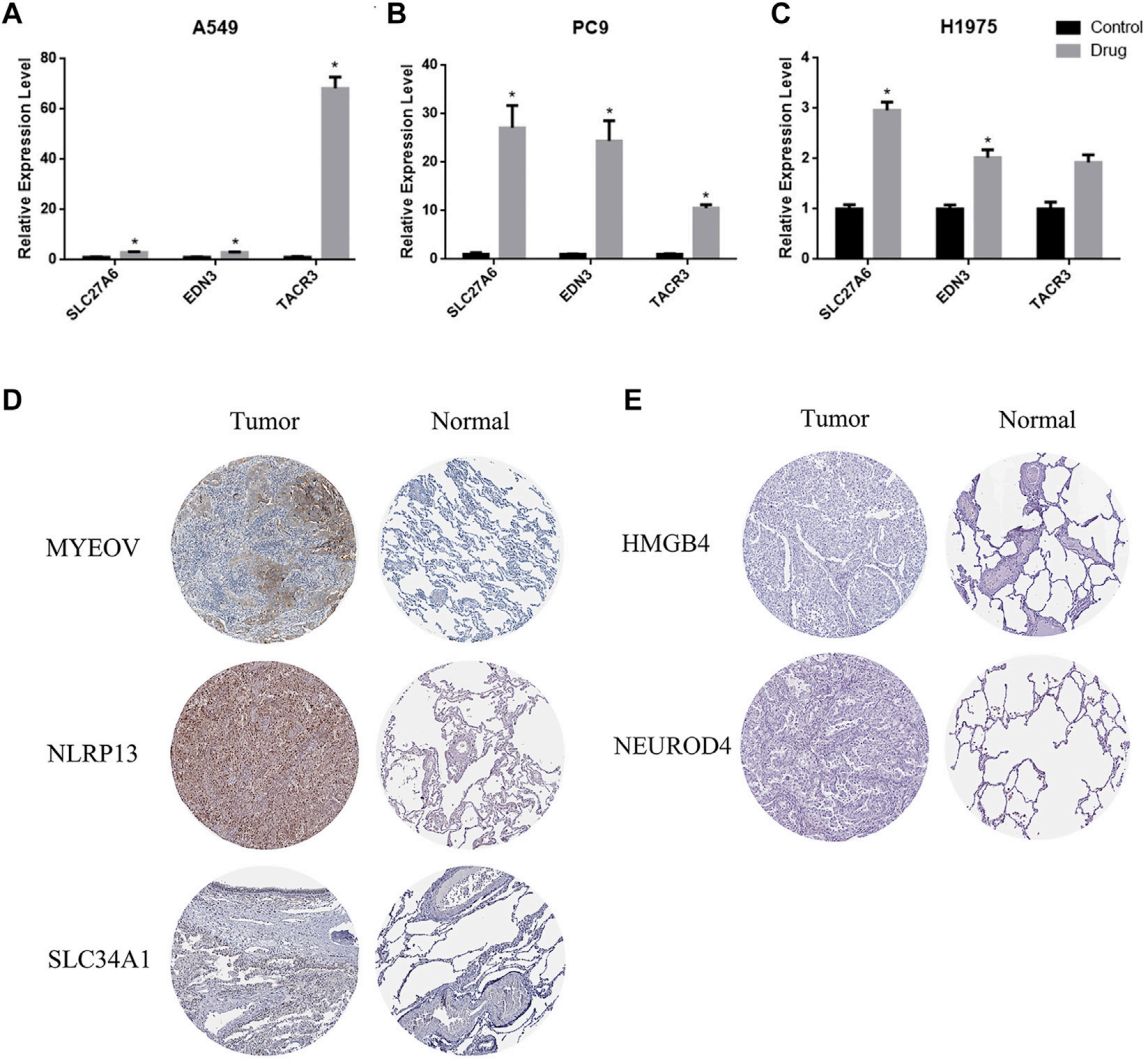
The expression of TACR3, EDN3 and SLC27A6 is related to promoter region methylation. **(A–C)** qPCR was used to detect the mRNA levels of TACR3, EDN3 and SLC27A6 in A549, PC9 and H1975 cells before and after the 5-Aza-2′-deoxycytidine treatment. **(D–E)** Immunohistochemistry images obtained from the HPA database demonstrated the protein expression of the 5 hypomethylated genes.

The prognostic methylation signature consists of 11 genes. Three of them are hypermethylated in LUAD (*TACR3*, *EDN3* and *SLC27A6*). We selected the broad-spectrum demethylation drug 5-aza-2′-deoxycytidine (5-aza) to treat LUAD cells (A549, PC9 and H1975) and measured the mRNA levels of *TACR3*, *EDN3* and *SLC27A6* by qPCR 4 days after treatment. The results showed that, compared with the control group, the transcription levels of these 3 genes were significantly increased after treatment with 5-aza (Figures 7A–C), suggesting that the transcriptional regulation of *TACR3*, *EDN3* and *SLC27A6* was related to promoter methylation. The other 8 genes showed low methylation and high mRNA expression in LUAD cells (*MYEOV*, *NLRP13*, *SLC34A1*, *NEUROD4*, *HMGB4*, *KCNU1*, *GABRA5* and *TRPM8*). Since there is no broad-spectrum drug to improve DNA methylation, we used the Human Protein Atlas (HPA) (https://www.proteinatlas.org/) to verify the expression of the proteins encoded by these genes in LUAD and normal tissues. Five of them (*MYEOV*, *NLRP13*, *SLC34A1*, *NEUROD4* and *HMGB4*) had protein expression data in the database, and *MYEOV*, *NLRP13* and *SLC34A1* were highly expressed in tumor tissues (Figure 7D), while *NEUROD4* and *HMGB4* showed no significant difference (Figure 7E).

## 4 Discussion

DNA methylation, as one of the most studied epigenetic alterations related to tumor phenotype, is of great significance for tumor research (Mazor et al., 2016; Biswas S Rao, 2017). Previous research showed that the overall DNA methylation pattern in tumor cell genomes is hypomethylation, while many CGIs associated with promoters showed focal hypermethylation (Hansen et al., 2011). Promoter hypermethylation was associated with tumor suppressor-related gene silencing, while the hypomethylation of the tumor cell genome could increase genomic instability (Feinberg and Vogelstein, 1983; Goelz et al., 1985; Pfeifer, 2018). Abnormal DNA methylation can be used not only as a target for tumor therapy but also as a biomarker for diagnosis and prognosis (Yamashita et al., 2018; Szejniuk et al., 2019). With the public information provided in TCGA, we conducted a comprehensive analysis and identified 11 methylation-related genes (*TACR3*, *SLC27A6*, *EDN3*, *TRPM8*, *MYEOV*, *NLRP13*, *KCNU1*, *NEUROD4*, *GABRA5*, *SLC34A1* and *HMGB4*) to predict the prognosis of LUAD. These genes not only were differentially methylated and expressed in LUAD tumor tissues from TCGA but also were related to the prognosis of patients. The survival curves showed that there was a significant difference in the survival curve between the higher-risk and lower-risk groups, especially in patients with early LUAD. Some of the 11 identified methylation-related genes have been shown to be abnormally expressed and important in cancer or other diseases. For example, *TRPM8* is a calcium permeability channel abnormally expressed in multiple malignant tumors. There is evidence that *TRPM8* plays a major role in promoting cell invasion and preventing replicative senescence (Yee, 2016). In the present study, *TRPM8* showed hypermethylation and low RNA expression in LUAD samples from TCGA, and the hypermethylation and low expression of *TRPM8* were associated with long survival. Previous studies have shown that decreased expression or inactivation of *EDN3* can inhibit the migration of cancer cells and improve survival (Wang et al., 2013; Kim et al., 2017). Our results revealed that *EDN3* was hypermethylated and expressed at low levels in LUAD, which was associated with longer OS. Another gene, *MYEOV*, is a region of cancer-associated genomic amplification. The amplification of this gene was reported to promote the progression of NSCLC pancreatic ductal adenocarcinoma and colorectal cancer (Lawlor et al., 2010; Fang et al., 2019). Subsequent mechanistic studies showed that the overexpression of *MYEOV* might be regulated by promoter hypomethylation (Liang et al., 2020). In accordance with the above results, our studies showed that *MYEOV* was hypermethylated and expressed at low levels in LUAD and that hypermethylation was positively correlated with survival time. As for other genes in the DNA methylation feature. TACR3 was found to be highly elevated in endometrial carcinoma. Although the role of *TAC1-TACR3* axis is not clear. Haixu et al. found that highly methylated *TAC1* promoted the development of endometrial carcinoma through the deregulation of *TAC* (Xu et al., 2018). Kyoichi Obata et al. found that *TACR3* protein showed significant and significant overexpression at the onset of bone matrix invasion in oral squamous cell carcinoma (Obata et al., 2016). *SLC27A6* is used as a predictor in the genetic analysis of colorectal cancer, prostate cancer, pancreatic cancer, and other tumors (Mohammed et al., 2019; Uhan et al., 2020; Verma et al., 2020; Zhong et al., 2021). However, basic research on this topic is still very limited. As a neuron differentiation factor, *NEUROD4* has been reported overexpressed in neuroendocrine tumors. Studies have shown that the continuous expression of *NEUROD4* in neuronal cells may be related to the regeneration of neural cells, and its expression level gradually decreases with the maturation of neurons (Masserdotti et al., 2015; Cecil et al., 2016). *SLC27A1* is rarely studied in tumors, but in recent years, some articles have pointed out that *SLC27A1* is highly expressed in melanoma and breast cancer and enhances tumor invasion, migration, and growth (Kwaepila et al., 2006; Zhang et al., 2018). The expression of *GABRA5*, which encodes the α *5-GABAA* receptor, has a synthetic lethal role in *MYC*-driven medulloblastoma (Sengupta et al., 2014). *NLRP13*, *KCNU1*, and *HMGB4*, although not as studied in tumors compared to the other genes in the model, need further exploration. Many articles verify the effectiveness of their own prediction formulas by comparing them with TNM staging (Peng et al., 2020; Zhu et al., 2021; Qiu et al., 2022). In our research, the DNA methylation feature score is an independent predictor and is not associated with the TNM stage. ROC curve analysis showed that the combination of the DNA methylation feature score and TNM stage was better for prognosis than TNM stage alone, suggesting that the combination of the 2 might help to improve the prediction of OS in LUAD patients. In addition, we found that the transcription levels of *TACR3*, *EDN3* and *SLC27A6* in LUAD cells were significantly increased by treatment with broad-spectrum demethylating drugs. These results suggested that the low expression of these genes was related to promoter hypermethylation. At the same time, the expression of some hypomethylated genes (*MYEOV*, *NLRP1*3 and *SLC34A1*)in immunohistochemical sections of LUAD was significantly stronger than that in lung tissues. These results showed that the identified genes are worthy of further study as biomarkers of methylation in LUAD.

Because of its noninvasive and fast characteristics, detecting circulating tumor DNA (ctDNA) in blood to monitor epigenetic changes in tumor DNA has become a very promising technology. Although this technology is not sufficiently mature, blood testing based on a single DNA methylation biomarker has been approved (Frankell and Jamal-Hanjani, 2022). Our study shows that the methylation signals of these 11 genes may be used as candidate markers to detect ctDNA methylation in LUAD patients. This model can predict the prognosis of patients with low cost and high efficiency.

However, our research still has several limitations. First, DNA methylation biomarkers are not effective in predicting advanced LUAD. Considering the small sample size of the advanced LUAD group, the results will have a certain deviation. Second, because it was difficult to obtain data with a sufficient sample size and consistent methylation detection platform, we did not use other datasets to verify the methylation formula. However, considering the large sample size of this study, this model was less likely to be an accidental feature of methylation noise but more likely to be a determinant of LUAD survival. Finally, our basic experiments were limited. We did not regulate the specific methylation site of genes. Further experimental studies on these genes will help to determine further their therapeutic potential.

## 5 Conclusion

In conclusion, we explored the characteristics of DNA methylation in LUAD. Furthermore, we confirmed a DNA methylation feature consisting of 11 genes. DNA methylation is associated with the survival of LUAD patients and can provide a better OS predictive ability when combined with TNM stage. Unfortunately, it was difficult for us to obtain a sufficient sample size and consistent methylation detection platform data to verify this methylation formula. However, our experiments indicated that the transcription of the hypermethylated genes was increased after demethylation with 5-aza, suggesting the validity of these results and indicating the potential value of these 11 genes in the study of LUAD prognosis.

## Data Availability

The original contributions presented in the study are included in the article/Supplementary Materials, further inquiries can be directed to the corresponding author.
