# Supplementary material for: An integrative analysis of DNA methylation and gene expression to predict lung adenocarcinoma prognosis
Source: Front Genet. 2022 Aug 29;13:970507. doi: 10.3389/fgene.2022.970507 (PMC9465336; doi:10.3389/fgene.2022.970507)

Fig.S1

The correlation between methylation level and gene expression of these 11 genes

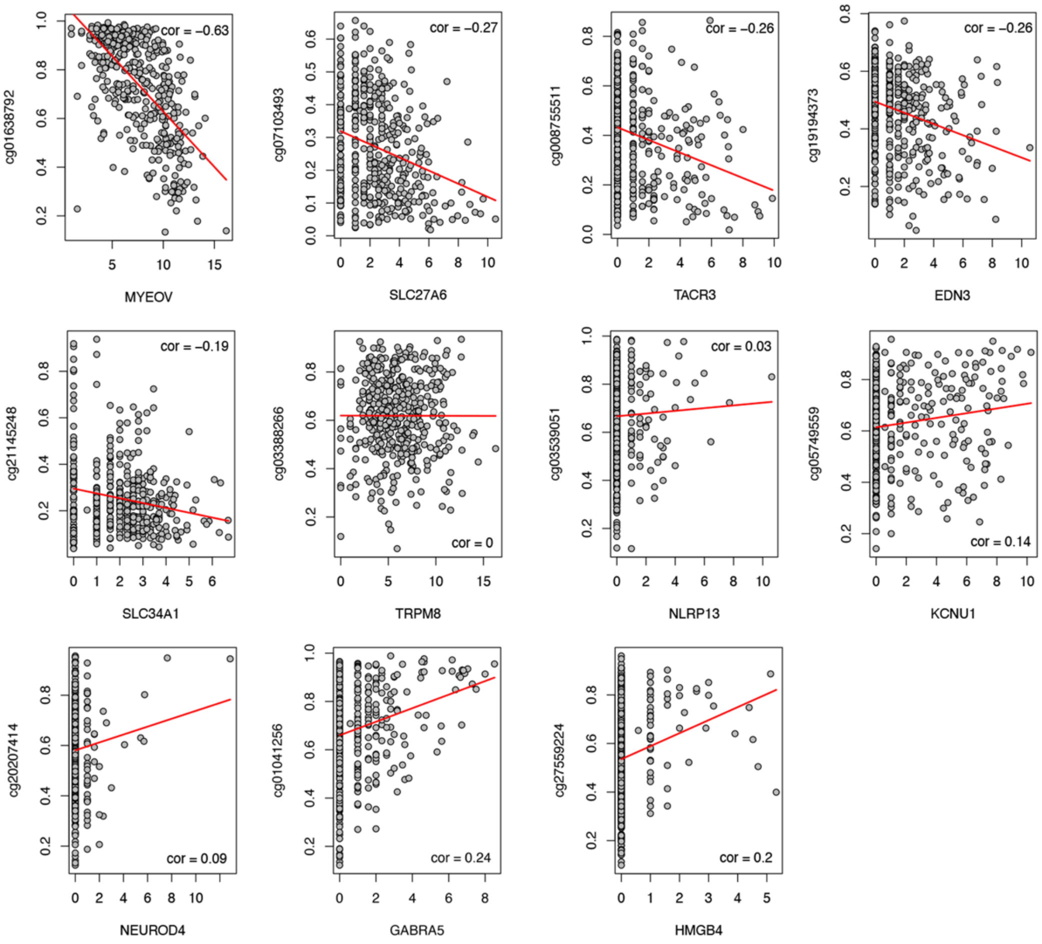

Fig.S2

The relationship between methylation feature scores and tumor immune infiltration.

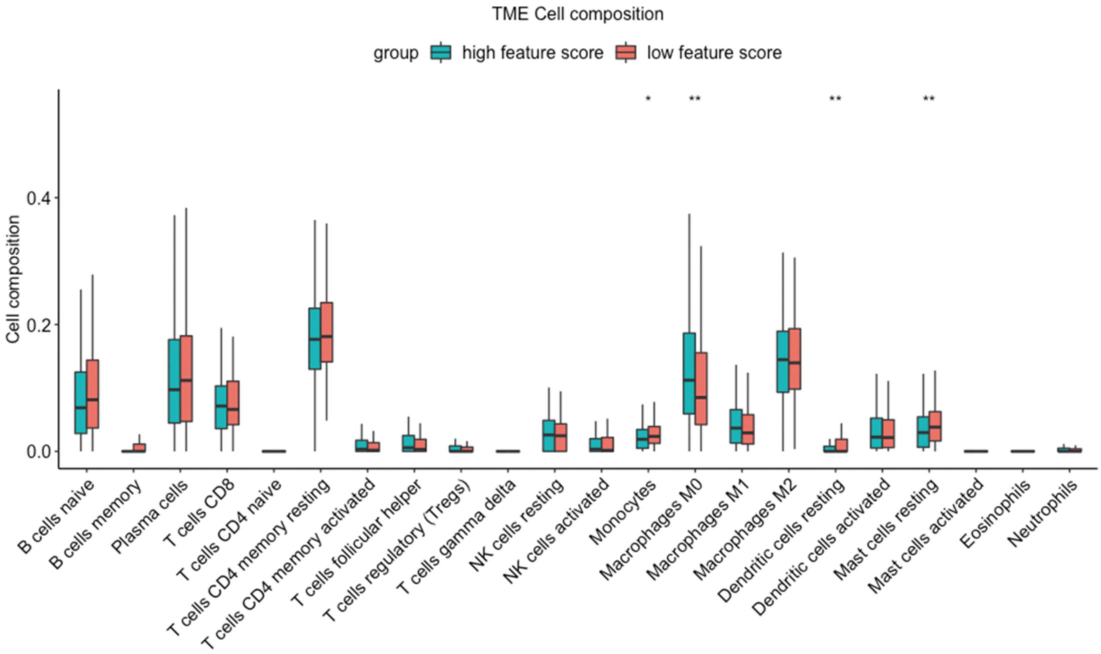

Supplement: Supplementary file 1 [file DataSheet1.PDF]
